# *Marsdenia tenacissima* extract overcomes Axl- and Met-mediated erlotinib and gefitinib cross-resistance in non-small cell lung cancer cells

**DOI:** 10.18632/oncotarget.18137

**Published:** 2017-05-24

**Authors:** Shu-Yan Han, Wei Zhao, Hai-Bo Han, Hong Sun, Dong Xue, Yan-Na Jiao, Xi-Ran He, Shan-Tong Jiang, Ping-Ping Li

**Affiliations:** ^1^ Key Laboratory of Carcinogenesis and Translational Research (Ministry of Education), Department of Integration of Chinese and Western Medicine, Peking University Cancer Hospital & Institute, Beijing 100142, PR China; ^2^ Key Laboratory of Carcinogenesis and Translational Research (Ministry of Education), Department of Cell Biology, Peking University Cancer Hospital & Institute, Beijing 100142, PR China; ^3^ Key Laboratory of Carcinogenesis and Translational Research (Ministry of Education), Department of Biobank, Peking University Cancer Hospital & Institute, Beijing 100142, PR China

**Keywords:** Marsdenia tenacissima extract, TKI resistance, NSCLC, Axl, c-Met

## Abstract

Tyrosine kinase inhibitors (TKIs) are an effective treatment strategy for non-small cell lung cancer (NSCLC) patients harboring mutations that result in constitutive activation of the epidermal growth factor receptor (EGFR). However, most patients eventually develop resistance to TKIs. This occurs due to additional EGFR mutations or the activation of bypass signaling pathways. In our previous work, we demonstrated that *Marsdenia tenacissima* extract (MTE) restored gefitinib sensitivity in resistant NSCLC cells with EGFR T790M or K-ras mutations. However, the potential efficacy of MTE in NSCLC cells with resistance mediated by Axl and c-Met, and the related molecular mechanisms need to be elucidated. In this study we evaluated the ability of MTE to restore erlotinib/gefitinib sensitivity in TKI resistant HCC827/ER cells and xenograft mice models. Our results demonstrate that MTE overcomes erlotinib and gefitinib resistance driven by Axl and c-Met *in vitro* and *in vivo*. Combination therapy significantly suppressed EGFR downstream molecules and the c-Met and Axl activated bypass signaling pathways. Moreover, we observed that MTE is more efficient at restoring resistance to erlotinib than gefitinib. As the Axl and c-Met mediated bypass pathways share the same downstream signaling cascade as EGFR, simultaneous targeting of these pathways is a promising strategy to overcome acquired resistance of TKIs. Our results demonstrate that MTE treatment attenuates Axl phosphorylation and the associated epithelial-mesenchymal transition, suggesting MTE treatment may be a potential therapeutic strategy for overcoming erlotinib and gefitinib cross-resistance in NSCLC, especially for erlotinib resistance.

## INTRODUCTION

Non-small cell lung carcinomas (NSCLC) commonly harbor oncogenic mutations in the EGF receptor (EGFR), resulting in constitutive activation [1]. In patients with these mutations, treatment with EGFR tyrosine kinase inhibitors (TKIs) such as erlotinib or gefitinib is a common and effective therapeutic strategy. The excellent response to these drugs in NSCLC cells expressing mutant alleles likely reflects their dependency on EGFR signaling for growth and survival [2, 3]. When the corresponding receptor tyrosine kinases (RTKs) are targeted by TKIs, this results in inhibition of key downstream signaling pathways such as the PI3K/Akt and MEK/ERK pathways, leading to cell growth arrest and death [4]. However, while these drugs are highly effective, the majority of patients treated with them eventually acquire TKI resistance [5–7].

The mechanisms underlying acquired resistance to EGFR-TKI treatment have been only partially elucidated. Resistance generally occurs through two main mechanisms, new, secondary, EGFR mutations activating the suppressed signaling pathways and activation of bypass signaling pathways [4]. The most common EGFR mutation leading to resistance is T790M, which accounts for 50% of EGFR-mutant lung cancers with acquired resistance [8]. Bypass signaling pathways result in resistance when additional receptors are activated, such as c-Met kinase [9], anexelekto (Axl) kinase [10], fibroblast growth factor receptor 1 (FGFR1) [11], and the NF-κB pathway [12]. These receptors share the same downstream signaling pathway as EGFR, resulting in pathway activation even in the absence of EGFR activity. Recent studies have indicated that elevated expression of the eukaryotic translation initiation factor 4E (eIF4E) and the epithelial-to-mesenchymal transition (EMT) are also linked to EGFR-TKIs resistance [13, 14]. While these events are behind many cases of EGFR-TKI resistance, the mechanisms underlying acquired resistance to EGFR-TKIs treatment are still unknown in over 40% of EGFR-mutant NSCLC patients.

Axl and hepatocyte growth factor (HGF) receptor c-Met are RTKs that share the same downstream pathways as EGFR, such as PI3K/Akt and MAPK/ERK [15, 16]. Axl is overexpressed in approximately 20% NSCLC patients exhibiting acquired resistance to EGFR-TKIs [17]. As signaling cross-talk between EGFR, Axl, and c-Met promotes metastasis and resistance to TKIs therapies [18, 19], combination treatment involving EGFR inhibitors and Axl or c-Met inhibitors is a promising new strategy for overcoming acquired resistance of EGFR-TKIs [20–22]. Studies have confirmed that simultaneous inhibition of Axl and c-Met by NPS-1034 [17] or AUY922 [22] is effective against EGFR-TKIs resistant NSCLC cells in *vitro* and *in vivo*. However, a phase II study of AUY922 and erlotinib did not meet its primary end-point due to the intolerable toxicities [23], and the efficacy of NPS-1034 in clinical trials remain to be determined.

The water extract of *Marsdenia tenacissima* (trade name: Xiao-Ai-Ping injection) has been approved to treat cancers by the China Food and Drug Administration for decades [24]. *M. tenacissima* extract (MTE) has been shown to enhance the clinical effects of chemotherapy against many cancers, including gastric cancer, lung cancer, and hepatocellular carcinoma [25, 26]. Studies show that pregnane derivatives are the principle components of MTE, and may contribute to its cytotoxic activities against cancer cells or its role in reversing drug resistance [27, 28]. Our previous work showed that treatment with MTE restored gefitinib sensitivity in resistant NSCLC cells with K-ras mutations or EGFR T790M mutation *in vitro* and *in vivo* [29, 30]. However, the potential efficacy of MTE on Axl and c-Met mediated resistance has not yet been investigated, and the related molecular mechanisms need to be defined.

The present study was performed in HCC827/ER cells, which was established by long-term exposure of parental HCC827 cells to erlotinib. HCC827/ER cells have have both c-Met amplification and Axl activation, and exhibit dual-resistance to gefitinib and erlotinib. We evaluated the effects of MTE on restoring gefitinib/erlotinib sensitivity *in vitro* and *in vivo* and explored the possible mechanisms.

## RESULTS

### Erlotinib-resistant HCC827/ER cells showed cross-resistance to gefitinib

**Figure 1:**
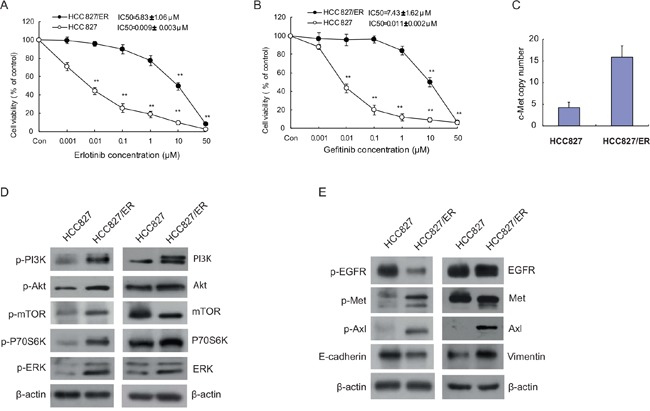
Cytotoxicity of EGFR-TKIs and molecular profiles in parental HCC827 and resistant cell line HCC827/ER Cells were treated with the indicated concentrations of erlotinib **(A)** and gefitinib **(B)** for 72 h in medium containing 1% FBS. Cell viability was determined using an MTT assay, and IC_50_ values were calculated using Graphpad Prism software 5.0. Results were expressed as the percentage of living cells compared to the control, error bars indicate SD of three independent measurements. *p <0.05, *p <0.01 *vs* control group. **(C)** The *Met* gene copy number of HCC827 and HCC827/ER cells was measured by real-time PCR using Taqman probes. **(D)** Basal expression of EGFR downstream signaling molecules in HCC827 and HCC827/ER cells was evaluated by Western blotting. **(E)** Protein expression of EGFR, bypass signal molecules c-Met and Axl, and epithelial-to-mesenchymal transition (EMT) markers in HCC827 and HCC827/ER cells. Protein (20 μg) from cell lysates was subjected to Western blot analysis. The results are representative of at least three independent experiments.

To assess the sensitivity of HCC827/ER cells and their parental cells HCC827 to erlotinib and gefitinib, both cell lines were exposed to 0.001 ∼ 50 μM erlotinib or gefitinib for 72 h. We then examined cell viability by MTT assay, and observed that HCC827 cells showed a dramatic decrease in cell viability compared with the HCC827/ER cells, indicating that HCC827/ER cell line is resistant to both erlotinib and gefitinib. As shown in Figure 1, HCC827/ER cells were 5000 times more resistant to erlotinib (Figure 1A) than HCC827 cells (IC_50_ = 5.83 μmol/L *vs* 0.009 μmol/L) and ≥ 7000 times more resistant to gefitinib (Figure 1B) than parental HCC827 cells (IC_50_ = 7.43 μmol/L *vs* 0.011 μmol/L).

### Mechanisms for acquired erlotinib resistance in HCC827/ER cells

We next sought to understand the mechanisms responsible for the observed EGFR-TKI resistance. Using a TaqMan qPCR assay, we showed that in accordance to past studies, HCC827/ER cells possess an increased c-Met copy number compared to the HCC827 parental cells (Figure 1C) [31]. Next, we examined changes in the EGFR signal transduction pathway and bypass signaling molecules in the resistant cell line HCC827/ER and their parental HCC827 cells by Western blotting. As shown in Figure 1D and 1E, compared with sensitive parental HCC827 cells, EGFR downstream pathway proteins PI3K, Akt, mTOR, and ERK were remarkably elevated in HCC827/ER cells (Figure 1D), as well as the bypass signaling pathway proteins phosphorylated c-Met, Axl, and phospho-Axl. These data confirm what was indicated by previous published reports (Figure 1E) [10]. Meanwhile, upregulated vimentin and downregulated E-cadherin also appeared in HCC827/ER cells compared to parental HCC827 cells (Figure 1E). Although decreased p-Met was observed in HCC827/ER cells after long-term erlotinib exposure (data not shown), the expression levels of p-Met were eventually upregulated when cultured for over 2 weeks in medium without erlotinib. As previous research indicated, the T790M mutation was not present in HCC827/ER cells [32].

### MTE restores erlotinib and gefitinib sensitivity in HCC827/ER cells

**Figure 2:**
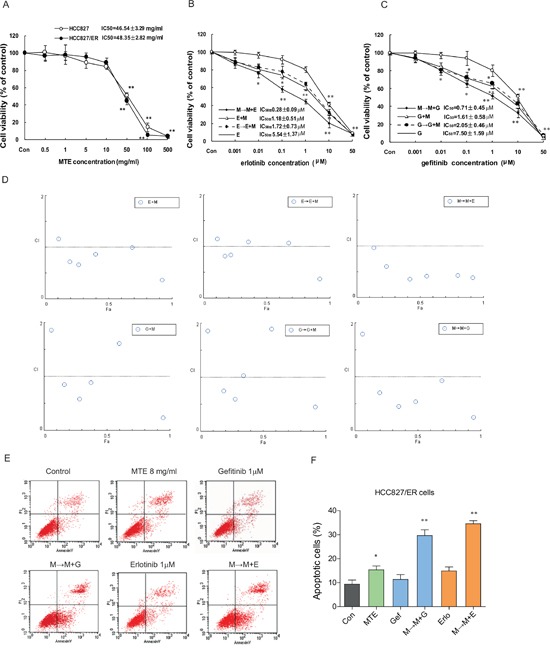
Effects of combined treatment of erlotinib or gefitinib with MTE on cell viability and apoptosis in HCC827/ER cells **(A)** The viability of parental HCC827 cells and resistant HCC827/ER cells after treatment with the indicated concentration of MTE for 72h. HCC827/ER cells were treated with erlotinib **(B)**, gefitinib **(C)**, or three different combinations of 8 mg/ml MTE and one of the other 2 drugs for 72 hours. Results were expressed as the percentage of living cells compared to the control, and error bars indicated SD of three independent measurements.*p < 0.05, *p < 0.01 *vs* control group. Combined effects were measured using the MTT assay and CI values **(D).** Cell apoptosis was determined by flow cytometry **(E)** after treatment with 1 μM erlotinib and gefitinib alone or their respective combinations with 8 mg/ml MTE (M→M+E for erlotinib, M→M+G for gefitinib) in HCC827/ER cells. **(F)** Quantitative results for Figure E. Each bar represents mean ± SD of three separate experiments. *p < 0.05, *p < 0.01 *vs* control group, ^#^*p* < 0.05 *vs* combinations of erlotinib or gefitinib with MTE. Con: control; G: gefitinib; E: erlotinib; MTE: *Marsdenia tenacissima* extract.

As we had shown that HCC827/ER cells are resistant to erlotinib and gefitinib, we next wanted to investigate the effect of erlotinib/gefitinib co-treatment with the natural anticancer drug MTE. We first used an MTT assay to examine the growth inhibitory effects of MTE 0.5 ∼ 500 mg/ml (equals to crude drug) alone in HCC827/ER and HCC827 cells. We found that MTE exerted similar IC_50_ values for these two cell lines, 46.54 ± 3.29 mg/ml for HCC827 and 48.35 ± 2.82 mg/ml for HCC827/ER respectively, indicating the efficacy of MTE is independent of the cellular sensitivity to erlotinib and gefitinib (Figure 2A).

Using a previously published equation for dosage calculation based on IC_50_ value [29], we set the concentration of MTE at 8.0 mg/ml (∼IC_15_) for further combination experiments. Next, we performed three sets of combination treatments of MTE and erlotinib or gefitinib; MTE pretreatment plus MTE/erlotinib combined treatment (M→M+E), no pretreatment plus MTE/erlotinib combined treatment (M+E), and erlotinib pretreatment followed by MTE/erlotinib combined treatment (E→M+E). The combination treatments procedures of MTE and gefitinib were set the same as erlotinib, including M→M+G, G+M, and G→G+M.

As previously demonstrated, MTE alone showed only weak suppression of cell growth. In Figure 2B, among the three combinations of MTE and erlotinib, we found M→M+E treatment was more potent over the other two procedures with an IC_50_ of 0.28 ± 0.09 μM, while E+M and E→E+M had a similar IC_50_ values of 1.18 ± 0.51 μM and 1.72 ± 0.73 μM, respectively. A similar result was observed upon the combined treatments of MTE and gefitinib on HCC827/ER cells, M→M+G treatment is the most potent one with an IC_50_ of 0.71 ± 0.45 μM, while M+G and G→G+M with an IC_50_ values at 1.61 ± 0.58 μM and 2.05 ± 0.46 μM, respectively (Figure 2C). Thus, we demonstrated that pretreatment with MTE restores sensitivity to erlotinib and gefitinib, resulting in remarkable proliferation inhibition in resistant cells. In addition, we found that MTE was more potent in overcoming erlotinib resistance, with a decreased effect seen when combined with gefitinib.

To help assess the potential combined effect of MTE and erlotinib/gefitinib and inform future treatment regimens, we calculated the combination index (CI). As demonstrated in Figure 2D, the M→M+E/G treatment was the most effective with a CI value of almost less than 1. These findings suggest that the synergistic effects of MTE and erlotinib or gefitinib were schedule-dependent as previously reported [29]. We also observed a more remarkable result when MTE combined erlotinib compared to gefitinib, suggesting MTE may be more effective at restoring erlotinib resistance in NSCLC.

### MTE pretreatment promotes erlotinib/gefitinib mediated HCC827/ER cell apoptosis

Compared with control groups, 1 μM erlotinib and gefitinib alone failed to trigger significant apoptosis in HCC827/ER cells, while MTE treatment caused apoptosis (16.38%, *P* < 0.05 *vs* control). Moreover, when MTE was combined with either erlotinib, or gefitinib, we observed significant apoptosis in HCC827/ER cells (Figure 2E). The M→M+E treatment induced significant apoptotic cell death (35.67%) (*P* < 0.01), and a similar result was also exerted by M→M+G treatment (30.86% apoptotic cells) (*P* < 0.01) (Figure 2F). The combination of M→M+E/G also showed a statistically significant induction of apoptosis when compared with each single drug (*P* < 0.05). These data reveal that the combination of MTE with erlotinib or gefitinib has a potent pro-apoptotic effect in the resistant HC827/ER cells.

### MTE and erlotinib/gefitinib combination treatment suppresses both EGFR downstream pathway and bypass activated c-Met and Axl

**Figure 3:**
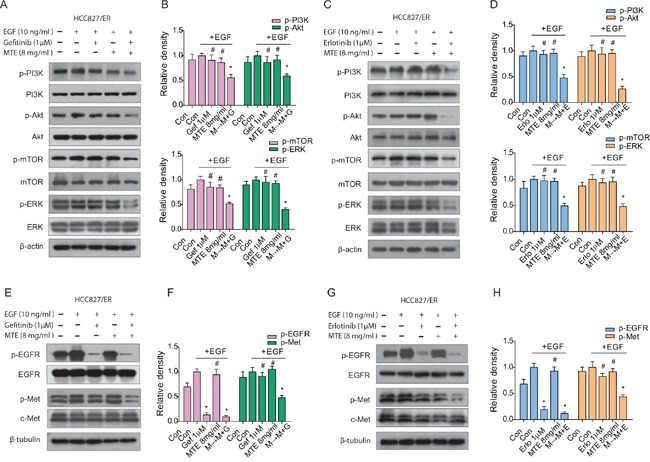
Effect of combined treatment of erlotinib or gefitinib with MTE on EGFR-related downstream molecules in HCC827/ER cells Cells were treated with 1 μM erlotinib or gefitinib alone or in combination with 8 mg/ml MTE (M→M+E for erlotinib, M→M+G for gefitinib) for 24 hours. Cells were stimulated with 10 ng/ml EGF for 15 min before harvest. Cell lysates were collected and subjected to SDS-PAGE and Western blotting, analyzed with phospho-specific antibodies to PI3K/Akt/mTOR, ERK1/2 (A for gefitinib, **C** for erlotinib), EGFR and c-Met (**E** for gefitinib, **G** for erlotinib). **(B and D)** are quantification of **(A and C)**, **(F and H)** are quantification of **(E and G)**, respectively. Each bar represents mean ± SD of three separate experiments. *p < 0.05 *vs* control group, ^#^*p* < 0.05 *vs* combinations of erlotinib or gefitinib with MTE. Con: control; Gef: gefitinib; Erlo: erlotinib; MTE: *M. tenacissima* extract; EGF: epidermal growth factor.

HCC827/ER cells were stimulated with 10 ng/ml EGF, activating the EGFR downstream signaling modulators PI3K/Akt/mTOR, ERK1/2 (Figure 3A and 3B for gefitinib, C and D for erlotinib), and c-Met (Figure 3E and 3F for gefitinib, G and H for erlotinib). Treatment with each drug by itself had no noticeable effect on expression of these EGFR-related molecules. However, the combined M→M+E and M→M+G treatments significantly inhibited the phosphorylation of PI3K/Akt/mTOR, ERK1/2, and c-Met, indicating the combinations are more effective than each drug alone at inhibiting the EGFR-downstream pathways (Figure 3B and 3F for gefitinib, D and H for erlotinib).

**Figure 4:**
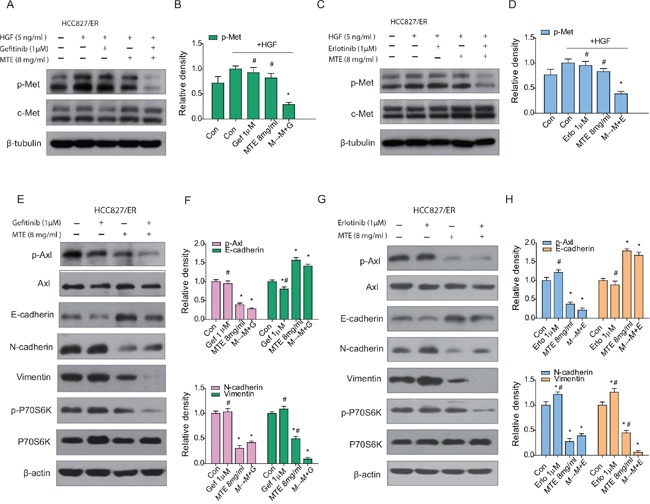
Effect of combined treatment of erlotinib or gefitinib with MTE on EGFR bypass signaling in HCC827/ER cells Cells were treated with 1 μM erlotinib and gefitinib alone or in combination with 8 mg/ml MTE (M→M+E for erlotinib, M→M+G fore gefitinib) for 24 hours. To determine the expression profile of p-Met, cells were stimulated with 10 ng/ml HGF for 5 min before harvest **(A-D).** Cells harvested for detection of other bypass signals were not stimulated with HGF **(E-H).** Cell lysates were collected and subjected to SDS-PAGE and Western blotting, analyzed with phospho-specific antibodies to p-Met (**A** for gefitinib, **C** for erlotinib), p-Axl, E-cadherin, N-cadherin, vimentin and p-P70S6K (**E** for gefitinib, **G** for erlotinib). **(B and D)** are quantification of the results in **(A and C)**, and **(F and H)** are quantification of **(E and G).** Each bar represents mean ± SD of three separate experiments. *p < 0.05 *vs* control group, ^#^*p* < 0.05 *vs* combinations of erlotinib or gefitinib with MTE. Con: control; Gef: gefitinib; Erlo: erlotinib; MTE: *M. tenacissima* extract; HGF: hepatocyte growth factor.

The aberrant activation of bypass signaling pathways such as c-Met and Axl are two important mechanisms that cause acquired resistance of EGFR-TKIs [15, 18]. HCC827/ER cells were stimulated with 5 ng/ml HGF to determine the changes of c-Met expression levels (Figure 4). As observed above, there was a significant downregulation of HGF activated c-Met after treatment with MTE combined with erlotinib or gefitinib (Figure 4A and 4B for gefitinib, C and D for erlotinib), while each drug alone had no obvious influence on phosphorylated Met expression. Axl is a receptor tyrosine kinase which is closely associated with the EMT phenotype [33]. EMT not only promotes a migratory phenotype in cells, but is also implicated in acquired resistance to TKIs [33]. Our results demonstrate that gefitinib treatment activates p-Axl and EMT in HCC827/ER cells (Figure 4E and 4F), while erlotinib treatment had a similar but more pronounced effect (Figure 4G and 4H). The combined M→M+E or M→M+G treatment did not alter the expression of Axl, but did effectively inhibit phosphorylation of Axl, preventing its activation. The combination treatments also decreased expression of the mesenchymal markers vimentin and N-cadherin, and increased the epithelial marker E-cadherin in HCC827/ER cells (Figure 4E and 4G). Surprisingly, MTE treatment alone also resulted in significant inhibition of p-Axl, as well as EMT transition. Meanwhile, the downstream product of PI3K/Akt/mTOR pathway, P70S6K was obviously reduced after combined M→M+E or M→M+G treatment (Figure 4E and 4G). These data suggest that MTE inhibits Axl phosphorylation and EMT transition in HCC827/ER cells, helping overcome erlotinib and gefitinib resistance.

### Combination treatment of MTE with erlotinib or gefitinib reduces tumor growth *in vivo*

**Figure 5:**
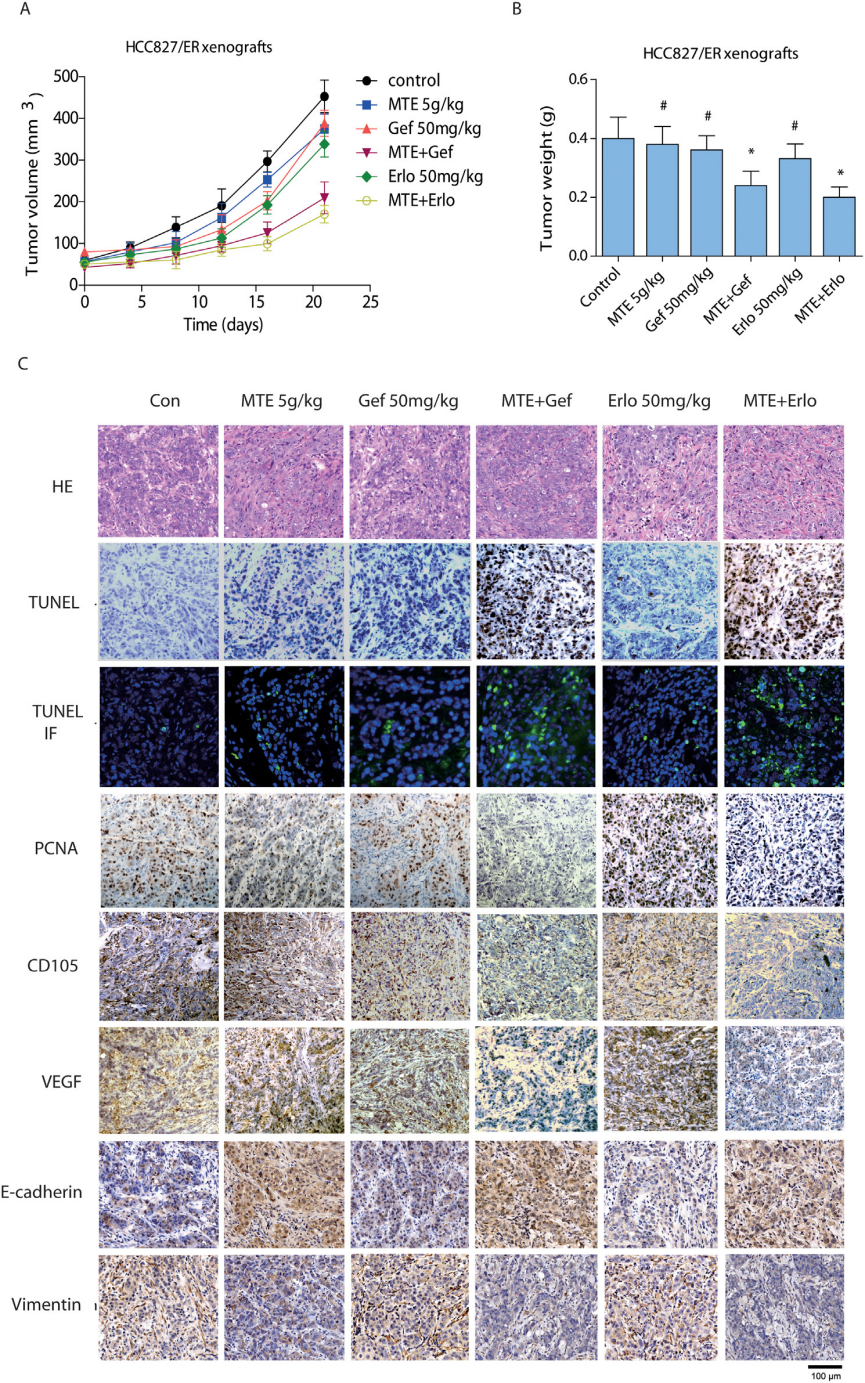
Effect of combined treatment of erlotinib or gefitinib with MTE on tumor growth, histological changes in HCC827/ER mice xenografts Tumor volume **(A)** and weight **(B)** of HCC827/ER xenografts was assessed. Mice were treated for 3 weeks with vehicle (control), gefitinib or erlotinib (50 mg/kg, p.o.), MTE (5 g/kg, i.p.), or combinations of MTE and one of the 2 other drugs as described in Materials and Methods. The weight of resected tumors was measured after animals were sacrificed. Histological changes **(C)** were detected by HE staining (200 ×) and immunohistochemistry staining to compare tumor growth (PCNA), cell apoptosis (TUNEL), tumor angiogenesis (CD105, VEGF), and EMT makers (E-cadherin, vimentin) between various treatment groups. Data are presented as the mean ± SE from mice in each group (n = 8). *p < 0.05 vs control group, ^#^*p* < 0.05 *vs* combinations of erlotinib or gefitinib with MTE. Con: control; Gef: gefitinib; Erlo: erlotinib; MTE: *M. tenacissima* extract.

To investigate the effects of combination treatment *in vivo*, we performed xenografts in nude mice followed by oral administration of MTE, erlotinib and gefitinib alone or in combination for 21 days. Our data showed that each of the drugs did not obviously inhibit tumor growth in HCC827/ER xenografts alone (Figure 5A and 5B). However, the tumor volume and tumor weight were remarkably reduced by the combined treatment of MTE and both TKIs (*P* < 0.01 *vs* control), especially when MTE was combined with erlotinib. Moreover, the two combination groups were both statistically significant compared to drug treatment alone (*P* < 0.05). These results indicate that MTE can restore the efficacy of TKIs such as erlotinib and gefitinib in NSCLC xenografts with c-Met and Axl mediated resistance. There was no obvious difference in the body weights of various groups (data not shown), but mice receiving EGFR-TKI treatment alone, especially the erlotinib group, displayed dry skin. There was no change in the behavior of mice before and after treatment.

### Effects of combined MTE with erlotinib or gefitinib on histological and immunohistochemical changes in mouse tumor tissues

As shown in Figure 5C, HCC827/ER xenograft tumor tissues isolated from mice treated with MTE combined with either erlotinib or gefitinib demonstrated remarkably decreased PCNA expressions. Apoptotic cells were detected by TUNEL assay with intense fluorescent-staining (green), and we observed a considerable increase in cell apoptosis with combined MTE and erlotinib or gefitinib treatment. The expressions of tumor angiogenesis markers, including VEGF and CD105, were dramatically decreased in the combination groups. Nevertheless, treatment of gefitinib, erlotinib, or MTE alone did not exert significant changes in expression of PCNA, cell apoptosis, or tumor angiogenesis. The expression of EMT-related markers were also changed by combination treatment in mice tumor tissues, as the level of E-cadherin was upregulated while vimentin expression was reduced. Interestingly, MTE alone also increased E-cadherin levels while suppressing vimentin expression in tumor tissues. These findings suggest that the antitumor activities of MTE and gefitinib or erlotinib combination therapy were mediated via the increase in tumor cell apoptosis, inhibition in tumor angiogenesis and EMT transition compared to the control group as well as each drug alone.

### Effects of MTE and erlotinib or gefitinib combination treatment on EGFR downstream pathways and bypass signaling pathways in xenograft tumors

**Figure 6:**
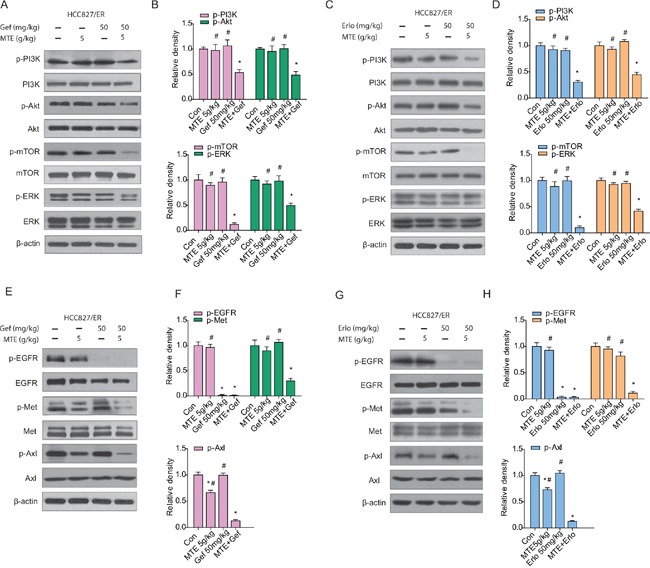
Effects of MTE and erlotinib or gefitinib combination treatment on EGFR downstream pathways and bypass signaling pathways in xenograft tumors Mice were treated as described previously. The resected tumors were lysed and total protein collected to measure EGFR related signaling molecules by Western blotting. The combined effects of MTE and erlotinib or gefitinib were evaluated through detecting the expression of EGFR downstream PI3K/Akt/mTOR and ERK (**A** for gefitinib, **C** for erlotinib), activation of bypass pathway markers c-Met and Axl (**E** for gefitinib, **G** for erlotinib). **(B and D)** are quantification of results found in **(A and C)**, and **(F and H)** are quantification of **(E and G).** Each bar represents mean ± SD of three separate experiments. *p < 0.05 *vs* control group, ^#^*p* < 0.05 *vs* combinations of erlotinib or gefitinib with MTE. Con: control; Gef: gefitinib; Erlo: erlotinib; MTE: *M. tenacissima* extract.

To understand the underlying mechanisms accounting for the impact of combination treatment on HCC827/ER tumor growth, we performed Western blotting to evaluate the associated signaling pathways. As demonstrated in Figure 6, each drug alone did not inhibit EGFR downstream pathways in HCC827/ER xenograft tumors (Figure 6A and 6B for gefitinib, C and D for erlotinib). However, we observed a remarkable reduction of p-PI3K, p-Akt, p-mTOR, and p-ERK expression levels in the combined group compared with each drug alone (*P* < 0.05) (Figure 6B and 6D). As expected, phosphorylation of EGFR was totally blocked by TKIs alone as well as when combined with MTE (Figure 6E and 6F for gefitinib, G and H for erlotinib).

Previous studies have indicated that c-Met and Axl signaling networks share the same downstream signaling pathways as EGFR, allowing cross-talk to occur between them [15, 16, 19]. In the present study, while the phosphorylation of c-Met and Axl were not significantly influenced by erlotinib or gefitinib treatment alone, they were considerably decreased in the groups that received MTE combined with erlotinib or gefitinib (Figure 6E-6H). Moreover, the combinations were more efficient than each agent alone at reducing c-Met phosphorylation and Axl activation (*P* < 0.05). Interestingly, MTE alone did not influence the phosphorylation of Met, but did reduce p-Axl expression in HCC827/ER tumors, which may contribute to the ability of combination therapy to overcome TKIs resistance.

## DISCUSSION

In this study, we evaluated the efficacy of MTE in overcoming Axl- and Met- mediated erlotinib and gefitinib resistance in NSCLC *in vitro* and *in vivo*. Our data demonstrate that MTE is effective in EGFR-TKI resistant HCC827/ER NSCLC cells through co-inhibition of the EGFR downstream pathways and the bypass signaling molecules dictated by Met or Axl.

Axl, which belongs to the TAM (Tyro-Axl-Mer) receptor tyrosine kinase family, is upregulated in NSCLC patients who have developed resistance to the first-generation EGFR TKIs gefitinib or erlotinib [34]. Axl activation has also been shown to cause imatinib resistance in gastrointestinal stromal tumors [35] and lapatinib resistance in HER2-positive breast cancer cells [36]. Moreover, Zhang et al. found that Axl was the most overexpressed gene in mouse tumors with acquired erlotinib resistance [10]. Axl activation can occur through its overexpression with or without the upregulation of its ligand GAS6 [10]. In EGFR-TKI resistant NSCLC, Axl can be transactivated by EGFR. Targeting Axl signaling can enhance the response to EGFR-TKI treatment and reverse acquired resistance in NSCLC [37]. Studies have indicated that upregulation of Axl and the subsequent EMT both lead to therapeutic resistance to TKIs in NSCLC [10, 33]. In the present study, we found that the levels of Axl and phosphorylated-Axl were significantly elevated in the resistant HCC827/ER cell line and its xenograft tumors. Moreover, the expression of mesenchymal markers vimentin and N-cadherin was elevated, while the epithelial marker E-cadherin was downregulated. Our data are consistent with prior studies in erlotinib resistant NSCLC cells [10] as well as studies in breast cancer cells demonstrating that the EMT phenotype is close associated with Axl overexpression [38].

In addition to Axl, the HGF/Met pathway is also frequently activated in EGFR-TKI resistant NSCLCs. Amplification of c-Met is considered one of the common causes of acquired resistance to EGFR-TKIs [10, 39]. While therapies against c-Met are initially effective in NSCLC patients, tumors eventually obtain acquired resistance as with other tyrosine kinase inhibitors [40]. Previous studies have indicated that Met and Axl are co-expressed in erlotinib resistant NSCLC, and that Axl activation is accompanied by increased Met expression [10, 21]. Consistent with these findings, our results demonstrate that upregulated Axl is concomitant with Met overexpression in the resistant HCC827/ER cells compared to the parental HCC827 cells. Axl and Met share overlapping downstream pathways with EGFR, and may trigger PI3K/Akt, MAPK/ERK, or NF-κB signaling to promote resistance to TKIs treatment in EGFR-mutant NSCLC [15, 16, 41]. Their cooperative and redundant actions suggest that co-targeting EGFR, Axl, and c-Met may achieve a desirable effect in TKI resistant NSCLC.

In the present study, we found that combination treatment with TKIs (erlotinib/gefitinib) and MTE, a Chinese herbal preparation that has been used in clinics for decades, causes a synergistic effect, inhibiting cell growth and enhancing cell apoptosis in TKI resistant HCC827/ER cell line. We observed that the downstream response to both EGF-induced PI3K/Akt/mTOR pathway activation and HGF-induced c-Met augmentation were suppressed by combined treatment with MTE and erlotinib or gefitinib. Interestingly, MTE alone was sufficient to remarkably downregulate Axl phophorylation, but without an obvious influence on other signaling molecules. Inhibition of p-Axl expression was further strengthened by the combined treatment of MTE and TKIs. Consist with the suppression of these signaling factors, the EMT of HCC827/ER cells was also significantly inhibited. We confirmed these *in vitro* effects of combined MTE and erlotinib or gefitinib treatment in a xenograft mouse model, and observed a reduction in tumor size and prevention of the side effects associated with erlotinib treatment such as skin peeling. Our results show that MTE is an effective treatment option for overcoming Axl- and Met- driven erlotinib or gefitinib resistance in HCC827/ER cells. We demonstrate that MTE functions through concurrently down-regulating EGFR, c-Met, and Axl, eventually resulting in the inhibition of Akt and ERK phosphorylation. However, further work will be necessary to determine whether Axl or c-Met is the primary target of MTE in overcoming EGFR-TKI resistance in NSCLC. Hopefully the efficacy of MTE in overcoming TKIs resistance in NSCLC will also be evaluated through clinical investigations.

The underlying mechanisms that cause acquired TKIs resistance are complicated, and there are multiple pathways by which tumors can escape from EGFR-TKIs targeted therapy. Each of the potential mechanisms is sufficient to sustain signaling that can drive tumor growth and survival through the constitutive activation of downstream pathways. This means that while targeting a single mechanism may restore TKI efficacy, it is easy for the tumor to develop resistance to inhibition of that pathway. Therefore, inhibition of multiple pathways is regarded as the most effective strategy to overcome TKI resistance in NSCLC.

In conclusion, we found that MTE could restore erlotinib and gefitinib efficacy in the resistant HCC827/ER NSCLC cell line with Axl and c-Met activation. These results suggest that the combination of MTE and TKIs may act as a potential therapeutic strategy for overcoming erlotinib and gefitinib cross-resistance in NSCLC.

## MATERIALS AND METHODS

### Drugs and reagents

Gefitinib (Iressa) was obtained from AstraZeneca (Cheshire, UK) and erlotinib (Tarceva) was purchased from Roche Pharma (Schweiz, UK). The stock solution was prepared in dimethyl sulfoxide (DMSO) at 20 mM and stored at −20°C. MTE (*M. tenacissima* extract, trade name: Xiao-Ai-Ping injection) (1g crude/ml) was obtained from SanHome Pharmaceutical Co., Ltd (NanJing, China). Our previous HPLC-MS analysis demonstrated that there were at least thirteen C-21 steroids in MTE [29]. Epidermal growth factor (EGF) and hepatocyte growth factor (HGF) were purchased from Biosource International Inc. (Camarillo, CA). Antibodies against p-PI3K, Akt, mTOR, p-mTOR (Ser2448), EGFR, p-EGFR (Tyr1068), Met, p-Met (Y1234/Y1235), Axl, p-Axl (Tyr702), ERK1/2, p-ERK1/2 (T202/Y204), E-cadherin, N-cadherin, vimentin, β-actin and β-tubulin were purchased from Cell Signaling Technology (Beverly, MA). PI3K and p-Akt (Ser473) were obtained from ABcam (Cambridge, UK).

### Generation of drug-resistant HCC827/ER cells and cell culture

Human NSCLC cell lines HCC827 and erlotinib-resistant HCC827 (HCC827/ER) were kindly provided by Professor Shi-Yong Sun (Emory University School of Medicine, Atlanta, GA). HCC827/ER cells were developed as described previously [14]. Cells were cultured in RPMI 1640 containing 5% fetal bovine serum at 37°C in a humidified atmosphere of 5% CO_2_. The HCC827/ER cells were maintained in the culture medium with 1 μM erlotinib for long term culture. Drug was removed at least 2 weeks prior to assay.

### Cell growth inhibition assay

The cell viability was measured by MTT assay. In brief, 1.5 × 10^4^ cells were plated in 96-well culture plates and incubated overnight. After that, cells were treated with gefitinib or erlotinib (0.001 ∼ 50 μM) or MTE (0.5 ∼ 500 mg/ml) for 72 h. The optical density at 570 nm was measured and the IC_50_ value was calculated based on the non-linear regression fit method by Graphpad Prism 5.0 software (San Diego, CA).

### Sequential combinations effect evaluation

Cells were plated in 96-well culture plates and incubated for 24 h, then treated by three different sequential combinations described previously [29]. Each sequential combination of MTE and gefitinib/erlotinib was characterized by a CI described by Chou [42] and calculated with CompuSyn (ComboSyn, Inc., Paramus, NJ, USA). CI value between 0.1-0.9 indicates synergism, 1.0-1.1 denotes additive, and > 1.1 means antagonism.

### Detection of cell apoptosis by flow cytometry

1×10^5^ cells were seeded in a six-well plate and incubated for 24 h. After that, cells were treated either with MTE only, gefitinib/erlotinib only or M→M+G/E as described above, respectively. Collected the cells and washed with phosphate-buffered saline (PBS), then labeled them with Annexin V and propidiumiodide (PI) (Biosea Biotechnology, Beijing, China). Cells were analyzed by flow cytometry (Bection Dikinson, USA) and assess the apoptotic rate by CellQest software.

### *Met* gene copy number analysis

The *Met* gene copy number was measured by real-time PCR using Taqman probes [43]. Genomic DNA was purified using the DNA easy Kit (Qiagen, Valencia, CA, USA). Primers, probes, and TaqMan Universal PCR reagent mix were purchased from Applied Biosystems (Foster City, CA, USA). *Met* gene amplification was determined in triple with 20 ng of genomic DNA. Data were normalized to RNase P and then calibrated to the normal human lung genomic DNA [44].

### Western blot analysis

Cells were lysed with RIPA lysis buffer, and the lysate was quantified by a bicinchonimic acid assay. The immunoblotting was carried out as described previously [29]. Briefly, cell lysate was separated by polyacrylamide gel electrophoresis (SDS-PAGE), followed by transfer onto PVDF membranes (Millipore, Bedford, MA, USA). The blots were blocked and probed with primary antibodies. Protein bands were visualized using an enhanced chemiluminescence kit, and their intensities were measured by ImageJ software. β-actin or β-tubulin was used as a loading control. Ratios of phosphorylated proteins to total proteins were calculated and normalized based on the corresponding control group.

### *In vivo* NSCLC tumor xenograft model

HCC827/ER (1.2 × 10^6^) cells with Matrigel (BD Biosciences) were subcutaneously inoculated into the right flank of male BALB/c nude mice. Tumor-bearing mice were randomly grouped (n=8 each) and treated as follows: vehicle control, orally administration of 50 mg/kg gefitinib or erlotinib, 5 g/kg MTE (i.p.), combined treatment of 5 g/kg MTE and 50 mg/kg gefitinib/erlotinib as previously described [30]. For the combination groups, MTE was given 12 h prior to the administration of gefitinib/erlotinib for the first time, and then simultaneously treated in the following days. Mice were terminated after 21 consecutive days of treatment and the tumors were collected. Tumor tissues were fixed in 10% buffered formalin for histological examination or cryopreserved for protein detection. The animal care and experiment protocols were performed in accordance with the Guidelines of Peking University Animals Research Committee.

### Histologic analysis and immunohistochemistry

Immunohistochemistry was performed as described in the previous study [30]. Paraffin-embedded tissue samples were cut at 5 μm thickness and mounted on positively charged glass slides. Each tissue block was stained with hemotoxylin and eosin. Tumor slides were incubated with primary antibody and visualized with 3,3-diaminobenzidine (DAB). Cells apoptosis was monitored using *In Situ* Cell Death Detection Kit, Fluorescein (Roche, Germany) and the apoptotic cells (green) were detected by a fluorescent microscope (Leica SP5, Germany).

### Statistical analysis

The data was presented as means ± SD or SE. All comparisons were analyzed with Student's two-tailed t test, with *P* < 0.05 considered statistically significant.

## Abbreviations

TKIs: tyrosine kinase inhibitors
RTKs: receptor tyrosine kinases
NSCLC: non-small cell lung cancer
EGFR: epidermal growth factor receptor
MTE: M*arsdenia tenacissima* extract
EMT: epithelial-to-mesenchymal transition
PI: propidiumiodide
eIF4E: eukaryotic translation initiation factor 4E
Axl: anexelekto
FGFR1: fibroblast growth factor receptor 1
M→M+E: MTE pretreatment plus MTE/erlotinib combined treatment
M+E: no pretreatment with MTE/erlotinib combined treatment
E→M+E: erlotinib pretreatment followed by MTE/erlotinib combined treatment
M→M+G: MTE pretreatment plus MTE/gefitinib combined treatment
M+G: no pretreatment with MTE/gefitinib combined treatment
G→M+G: gefitinib pretreatment followed by MTE/gefitinib combined treatment
CI: combination index
DMSO: dimethyl sulfoxide
EGF: epidermal growth factor
HGF: hepatocyte growth factor
DAB: 3,3-diaminobenzidine.
